# Structure-Based Discovery of Orthosteric Non-Peptide GLP-1R Agonists via Integrated Virtual Screening and Molecular Dynamics

**DOI:** 10.3390/ijms26136131

**Published:** 2025-06-26

**Authors:** Mansour S. Alturki, Reem A. Alkhodier, Mohamed S. Gomaa, Dania A. Hussein, Nada Tawfeeq, Abdulaziz H. Al Khzem, Faheem H. Pottoo, Shmoukh A. Albugami, Mohammed F. Aldawsari, Thankhoe A. Rants’o

**Affiliations:** 1 Department of Pharmaceutical Chemistry, College of Pharmacy, Imam Abdulrahman Bin Faisal University, P.O. Box 1982, Dammam 31441, Saudi Arabia; msmmansour@iau.edu.sa (M.S.G.); nztawfeeq@iau.edu.sa (N.T.); ahalkhzem@iau.edu.sa (A.H.A.K.); 2 Department of Pharmaceutical Sciences, College of Pharmacy, King Saud Bin Abdulaziz University for Health Sciences, Ministry of National Guard Health Affairs (MNGHA), Riyadh 11481, Saudi Arabia; khodierr@ksau-hs.edu.sa; 3 King Abdullah International Medical Research Center (KAIMRC), Ministry of National Guard Health Affairs (MNGHA), Riyadh 11481, Saudi Arabia; 4 Department of Pharmacology, College of Pharmacy, Imam Abdulrahman Bin Faisal University, P.O. Box 1982, Dammam 31441, Saudi Arabia; dahussein@iau.edu.sa (D.A.H.); fhpottoo@iau.edu.sa (F.H.P.); 5 College of Pharmacy, Imam Abdulrahman Bin Faisal University, P.O. Box 1982, Dammam 31441, Saudi Arabia; 2220007016@iau.edu.sa; 6 Department of Pharmaceutics, College of Pharmacy, Prince Sattam Bin Abdulaziz University, Al-kharj 11942, Saudi Arabia; moh.aldawsari@psau.edu.sa; 7 Department of Pharmacology and Toxicology, College of Pharmacy, University of Utah, Salt Lake City, UT 84112, USA; thankhoe.rantso@pharm.utah.edu; 8 Huntsman Cancer Institute, University of Utah, Salt Lake City, UT 84112, USA

**Keywords:** GLP-1R, virtual screening, molecular dynamics, oral therapy, chemical scaffolds

## Abstract

The development of orally bioavailable non-peptidomimetic glucagon-like peptide-1 receptor agonists (GLP-1RAs) offers a promising therapeutic avenue for the treatment of type 2 diabetes mellitus (T2DM) and obesity. An extensive in silico approach combining structure-based drug design and ligand-based strategies together with pharmacokinetic properties and drug-likeness predictions is implemented to identify novel non-peptidic GLP-1RAs from the COCONUT and Marine Natural Products (CMNPD) libraries. More than 700,000 compounds were screened by shape-based similarity filtering in combination with precision docking against the orthosteric site of the GLP-1 receptor (PDB ID: 6X1A). The docked candidates were further assessed with the molecular mechanics MM-GBSA tool to check the binding affinities; the final list of candidates was validated by running a 500 ns long MD simulation. Twenty final hits were identified, ten from each database. The hits contained compounds with reported antidiabetic effects but with no evidence of GLP-1 agonist activity, including hits **1**, **6**, **7**, and **10**. These findings proposed a novel mechanism for these hits through GLP-1 activity and positioned the other hits as potential promising scaffolds. Among the studied compounds—especially hits **1**, **5**, and **9**—possessed strong and stable interactions with critical amino acid residues such as TRP-203, PHE-381, and GLN-221 at the active site of the 6X1A-substrate along with favorable pharmacokinetic profiles. Moreover, the RMSF and RMSD plots further suggested the possibility of stable interactions. Specifically, hit **9** possessed the best docking score with a ΔG_bind value of −102.78 kcal/mol, surpassing even the control compound in binding affinity. The ADMET profiling also showed desirable drug-likeness and pharmacokinetic characteristics for hit **9**. The pipeline of computational integration underscores the potential of non-peptidic alternatives in natural product libraries to pursue GLP-1-mediated metabolic therapy into advanced preclinical validation.

## 1. Introduction

Type 2 diabetes (T2D) and obesity are among the most critical challenges faced by global public health. The International Diabetes Federation (IDF) has projected that the number of individuals suffering from diabetes worldwide may reach 783.2 million in 2045 [1]. Moreover, diabetes will be positioned as the seventh leading cause of mortality across the globe by the year 2030 [2]. The worldwide rise in diabetes mellitus (DM) is predominantly fueled by increasing obesity levels. Recent research demonstrates that global obesity rates have nearly tripled over the past 40 years, a trend that has been closely linked to a significant uptick in the occurrence of T2D [3].

**Figure 1:**
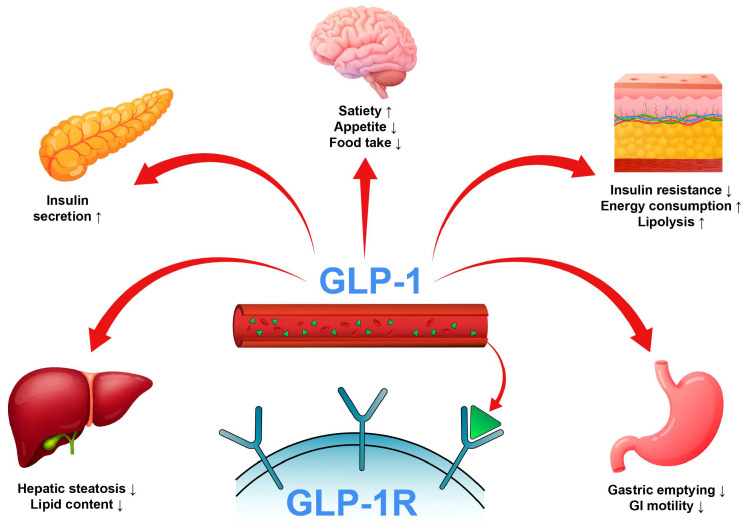
The comprehensive antidiabetic effects of GLP-1 signaling.

In the management of T2D and obesity, glucagon-like peptide-1 (GLP-1) receptor agonists (GLP-1RAs) have evolved into cornerstone therapy [4]. These agents augment insulin secretion, inhibit glucagon release, slow gastric emptying, and increase satiety; thus, they change the face of treatment for diabetes (Figure 1) [5]. As important as the discovery of the GLP-1 receptor (GLP-1R), which was more than 30 years ago, therapeutic agents directed toward this receptor bring astonishing effects in aspects such as glycemic control and weight reduction. The first success of peptide-based GLP-1RAs, exenatide, increased interest in other injectable formulations when further improvements were made to diabetes treatment [6]. Since then, a limited number of GLP-1 RAs have received approval from the U.S. Food and Drug Administration (FDA) for the treatment of T2DM and, in certain cases, for the management of obesity. Among these, liraglutide, semaglutide, and dulaglutide stand out as some of the most recognized agents [7,8,9]. All these are synthetic peptide-based analogs of GLP-1 [10]. For both weight management and diabetes control, only semaglutide and liraglutide have received FDA approval [11,12,13].

**Figure 2:**
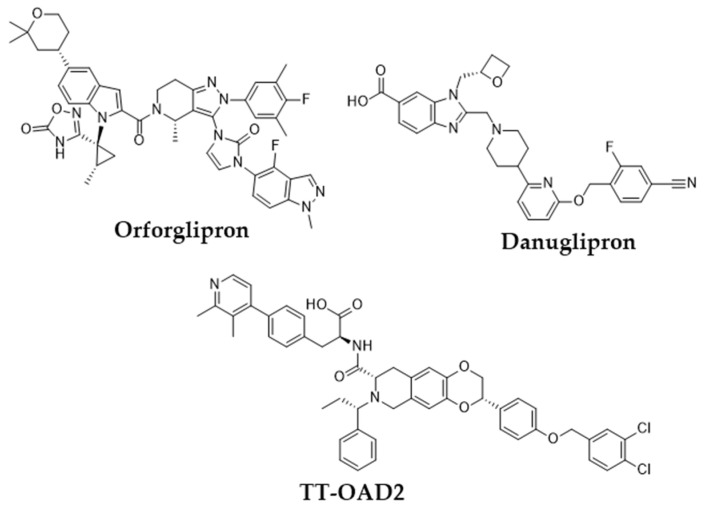
Examples of non-peptide GLP-1R agonists.

However, peptide-based therapies have major drawbacks, the most notable being their administration by injection, which affects compliance and convenience for patients [14,15]. This is where the research focus has shifted toward developing orally bioavailable GLP-1RAs. The oral administration route is expected to significantly improve patient compliance due to the reduced burden associated with injectable therapies. Transitioning from peptide-based therapies to non-peptide agonists (NPAs) is a promising avenue; however, developing small molecules replicating endogenous GLP-1 pharmacokinetics remains challenging [14]. Recent advancements in cryogenic electron microscopy (cryo-EM) promise insights into the structural activation of GLP-1R have also led the way in discovering new classes of non-peptide GLP-1R agonists [15,16]. Compounds such as orforglipron, danuglipron, and TT-OAD2 have shown promising pharmacokinetic profiles, and early clinical results suggest that they could be an efficient and practical alternative to existing peptide oral GLP-1RAs (Figure 2) [17,18,19,20,21,22,23]. The breakthrough in oral delivery was semaglutide, the first oral GLP-1RA approved in 2019 by the FDA for the management of T2DM, which represented a huge milestone in this area [24]. It has been shown that oral semaglutide is effective in lowering hemoglobin A1c (HbA1c) levels and in weight loss, like the results of other routes [24]. This demonstration gave extra credence to the therapeutic potency of oral GLP-1RAs and further motivated research into small molecules that would mimic the effects of peptide-based GLP-1RAs. One among those is cinchonine, which has been presented as a promising contender with anti-hyperglycemic effects and benefits in obesity-affiliated conditions like non-alcoholic steatohepatitis (NASH) [25]. Indeed, the small molecules derived from various discoveries, yet with similar pharmacological profiles to peptide-based GLP-1RAs, but amenable to oral administration, usher in new hope for treating T2D and obesity. Important pharmacokinetic modifications targeting specific carriers and surface coatings will ultimately improve the bioavailability and stability of GLP-1RAs when administered orally. Such designs fundamentally counteract challenges usually associated with peptide medications, including poor intestinal absorption and enzymatic degradation within the gastrointestinal tract [26]. However, apart from hyperglycemia control, GLP-1RAs have cardiovascular benefits and protection for renal and hepatic organs, which would further build on the therapeutic interest of patients suffering from comorbid conditions such as chronic kidney disease and non-alcoholic fatty liver disease (NAFLD) [27,28]. Yet despite these advances, an apparent void exists in identifying novel non-peptide GLP-1-RAs featuring optimized pharmacokinetic profiles regarding effectiveness and safety for long-term T2DM and obesity treatment. Although many small-molecule agonists have shown some promise, a need for intense evaluation of alternative sources for new GLP-1RAs exists.

Natural products (NPs) are accessible and cost-effective sources that can help solve the challenge of identifying an oral GLP-1 therapeutic agent. The COCONUT and Comprehensive Marine Natural Products (CMNPD) databases, with their vast chemical space and diverse bioactivity, offer a vast, understudied ground for discovering novel non-peptide GLP-1RAs. Such compounds have demonstrated therapeutic promise in disease areas such as metabolic disturbances and may be particularly well-suited to offer distinct advantages in structural diversity and pharmacological activity [29,30,31,32]. In this study, we employed an integrative molecular modeling strategy to predict candidate hits capable of binding to the orthosteric sites of GLP-1R. By screening a large and diverse library of natural and marine compounds, our approach demonstrates a potentially unbiased and powerful strategy for the discovery of novel non-peptide GLP-1 receptor agonists (GLP-1RAs).

## 2. Results and Discussion

Using integrated structure-based and ligand-based methods, two different natural product databases, including COCONUT and CMNPD, were screened for GLP-1R activation. To identify potential candidates as well as structural scaffolds for GLP-1R modulation, a combined virtual screening and molecular dynamics protocol was subsequently carried out. Marine and natural compounds with known antidiabetic activity that might activate GLP-1 were also evaluated in the results.

### 2.1. Database Preparation for Virtual Screening

#### 2.1.1. Physicochemical Parameters and Drug-Likeness

Finding GLP-1 agonists in both natural and marine sources is the primary goal of this endeavor. Therefore, a filtering technique is developed, emphasizing the calculation of drug-like properties. Several rules and principles that describe the physicochemical properties of a target drug, such as the rule of 3, the rule of 5, the Zinc, CNS, and respiratory drug-likeness principles, are combined with a statistical analysis of approved drugs to create the quantitative estimation of the drug-likeness (QED) model developed in FAFDrugs4 [33,34]. The filter thresholds were validated when it was discovered that the obtained chemical space could retain up to 90% of the oral medications. The COCONUT database is an extensive library of natural products (NPs) gathered from public databases [35]. It includes 63 NP databases, such as Zinc NPs, NPAtlas, PubChem NPS, NCI developing medicines, and ChemSpider NPs. In its most recent version, this massive database contains 695,133 NPs. Additionally, the CMNPD provides a plethora of compound scaffolds covering a vast chemical space with intriguing possibilities for medicinal chemistry. Specifically, the database comprises approximately 47,000 compounds isolated from various marine organisms [36]. The databases were successfully downloaded from the COCONUT and the CMNPD websites and then further filtered using Schrödinger’s Canvas by removing duplicate structures followed by physicochemical descriptors originally included in the database, such as MW 100–600, logP −3–6, number of rings <6, total number of heavy atoms <50, total charge −4–4, number of H-donors <7, number of H-acceptors <12, number of rotatable bonds <11, and another total charge condition of −4–4 [37]. The QED incorporates eight physicochemical characteristics—including molecular weight, LogP, H-bond donors, H-bond acceptors, charge, aromaticity, stereochemistry, and solubility—into a score ranging from 0 to 1. Therefore, a molecule will appear more drug-like the closer its QED score is to 1 [34,38].

#### 2.1.2. Shape-Based Analysis

The GLP-1 co-crystallized ligand was screened against the filtered COCONUT database, which had 40,332 natural products, and the CMNPD, which contained over 12,000 marine compounds, using the shape-screening feature of Schrödinger’s software (Versions 2024-4 and 2025-1). A cutoff for shape similarity indices ≥0.3 was used to refine the shape screening results [39]. Following this procedure, 33,227 natural products and 1306 marine compounds were chosen for further virtual screening against the GLP-1R binding pocket.

### 2.2. Virtual Screening

**Figure 3:**
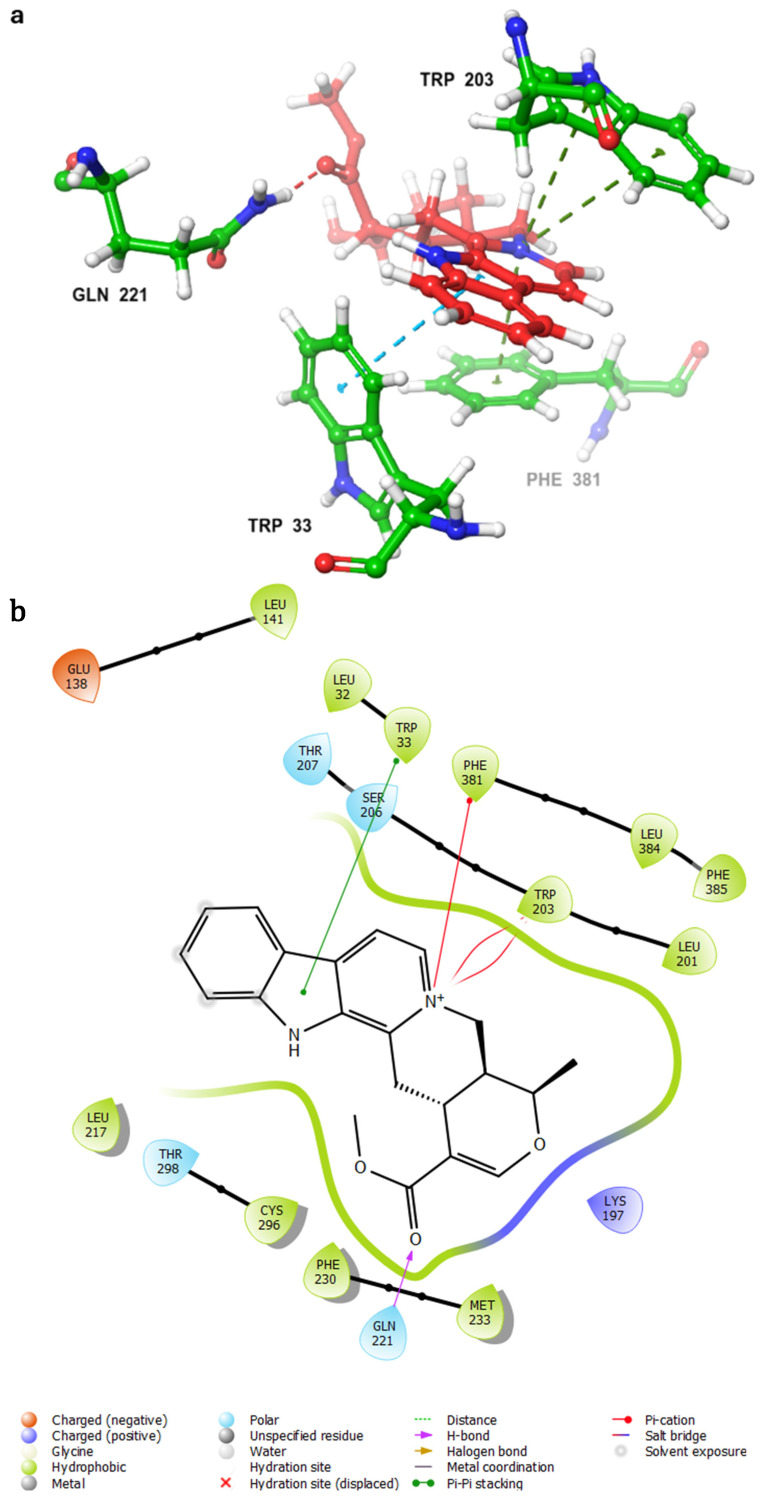

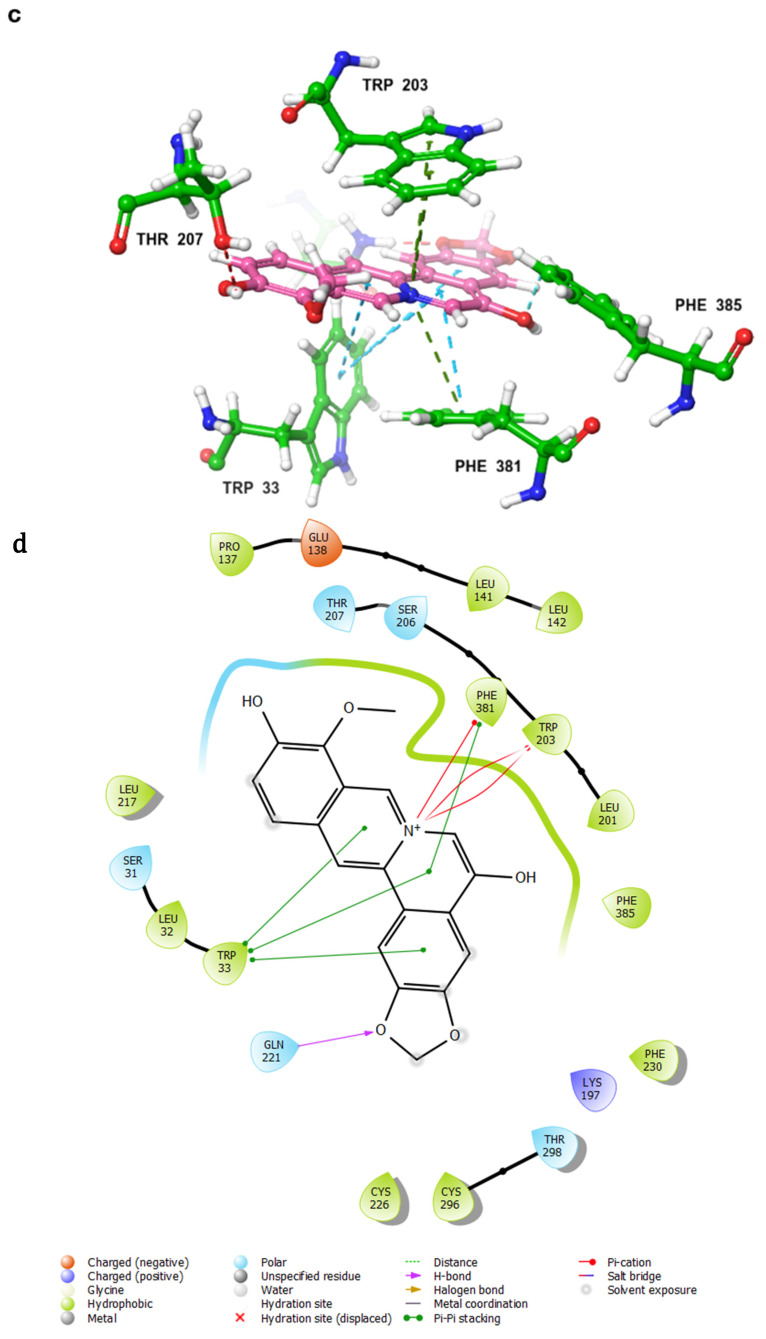

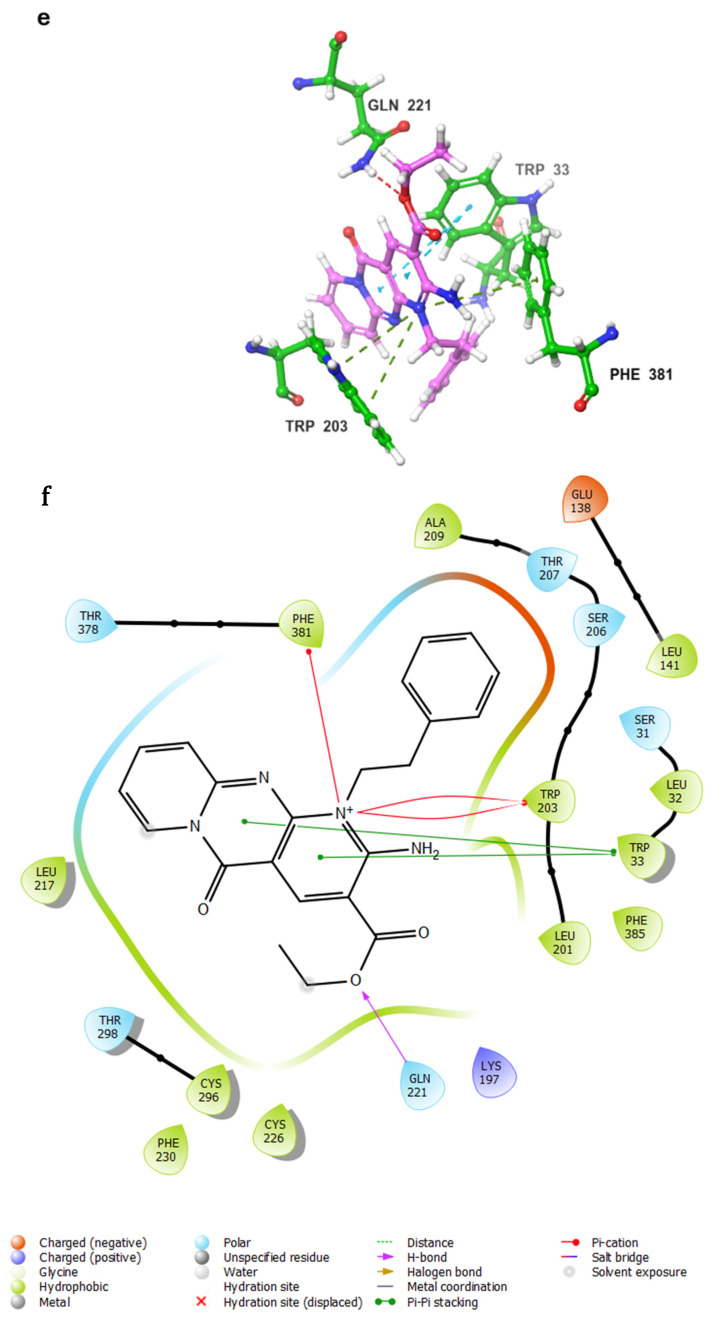

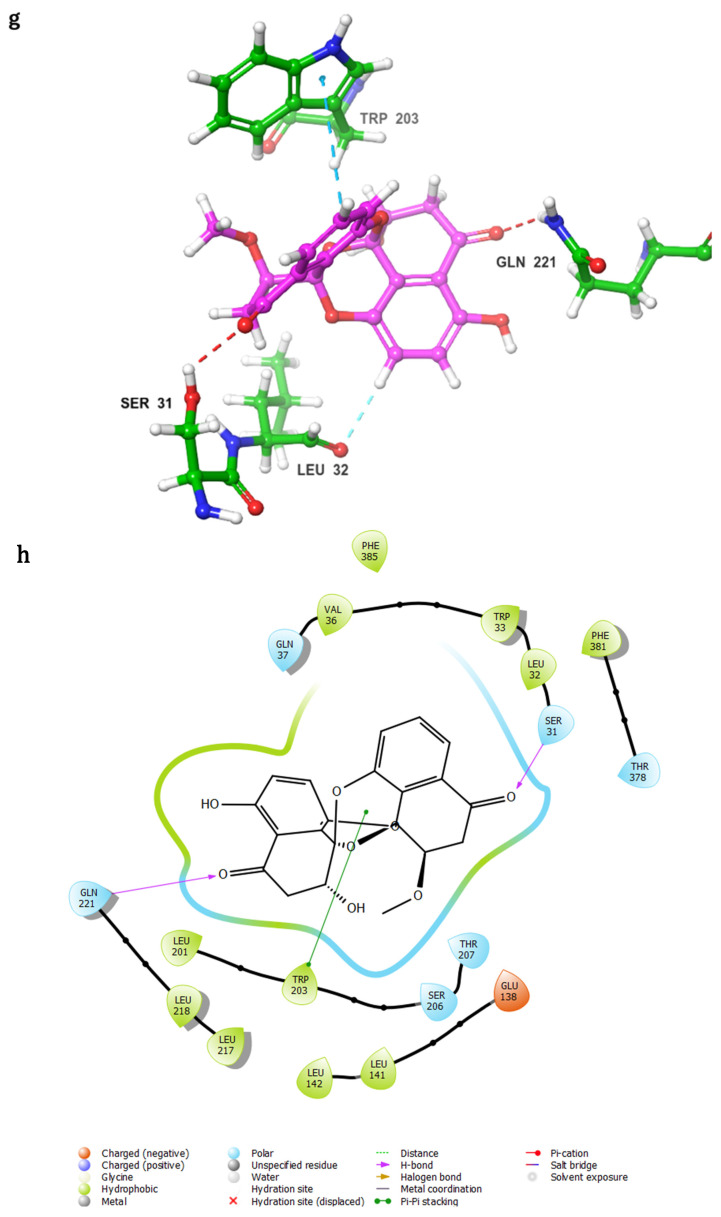

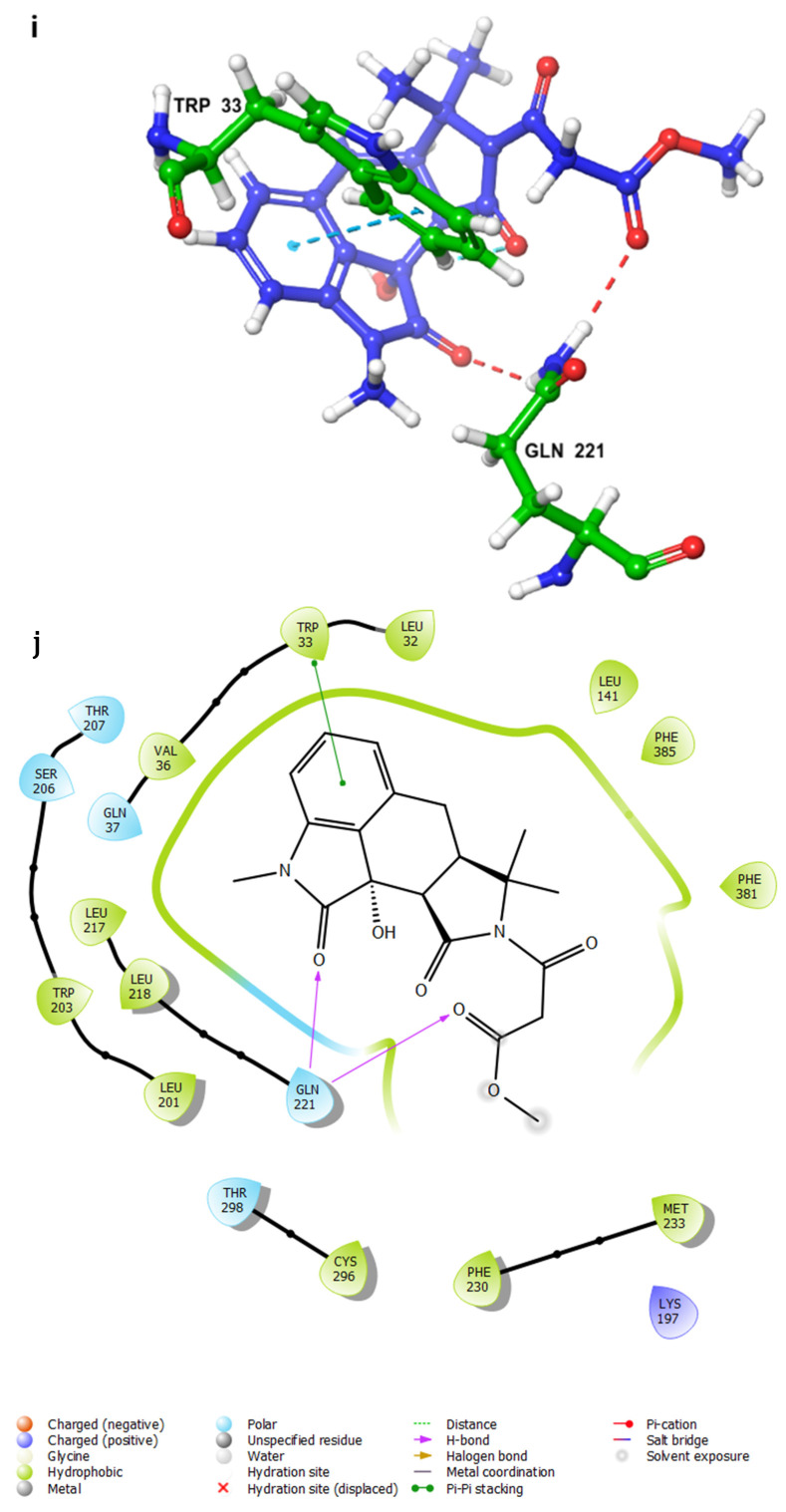
(**a**,**b**) 3D and 2D representation of the binding interactions between **1** and GLP1 (PDB ID: 6X1A). Ligand atoms are shown as sticks (carbon atoms colored in red), and the key residues are shown as sticks (carbon atoms colored in green). (**c**,**d**) 3D and 2D representation of the binding interactions between **5** and GLP1 (PDB ID: 6X1A). Ligand atoms are shown as sticks (carbon atoms colored in salmon), and the key residues are shown as sticks (carbon atoms colored in green). (**e**,**f**) 3D and 2D representation of the binding interactions between **9** and GLP1 (PDB ID: 6X1A). Ligand atoms are shown as sticks (carbon atoms colored in magenta), and the key residues are shown as sticks (carbon atoms colored in green). (**g**,**h**) 3D and 2D representation of the binding interactions between **11** and GLP1 (PDB ID: 6X1A). Ligand atoms are shown as sticks (carbon atoms colored in plum), and the key residues are shown as sticks (carbon atoms colored in green). (**i**,**j**) 3D and 2D representation of the binding interactions between hit **13** and GLP1 (PDB ID: 6X1A). Ligand atoms are shown as sticks (carbon atoms colored in blue), and the key residues are shown as sticks (carbon atoms colored in green).

The resultant databases underwent three steps of virtual screening in Schrödinger’s Glide: high throughput virtual screening (HTVS), standard precision (SP), and extra precision (XP) docking [40]. Ten percent of the top-scoring compounds were retained in each phase, while the scoring hierarchy was maintained due to the protocol’s flexible docking approach, which included post-docking energy minimization. The final output following the XP docking stage included 276 and 36 compounds from the COCONUT and Marine databases, respectively. The XP scores of the top 10 hits were correlated with their binding energies (MM-GBSA) (Figure 3). The docking methodology was first verified by calculating the root mean square deviation (RMSD) of the crystal and docked poses of the co-crystallized ligand, resulting in 2.03 Å, indicating that Glide can predict binding to the GLP-1R binding site with high accuracy. PF-06882961 was also redocked successfully into its native receptor (6X1A) with RMSD 2.03 Å and GlideScore of −12.040. Cross-docking to non-native GLP-1R structures (6X19 and 6ORV) yielded lower RMSD values (1.15 Å and 1.19 Å), indicating higher pose agreement and demonstrating receptor pocket similarity as well as method robustness. GlideScores were slightly less favorable in cross-docked poses but were still indicative of strong binding (Table 1). These results demonstrate the strengths of ensemble-based docking methods and the limitations of one-pose redocking evaluation.

The results of the top hits from the COCONUT and Marine databases, as shown in Table 2 and Table 3, provide valuable insights into how well each compound binds to the GLP-1 receptor (PDB ID: 6X1A) through the descriptors of XP Score, MM-BBSA dG_Bind, and Shape Similarity. In the COCONUT database, the top hits displayed XP scores ranging from −13.835 to −12.532, indicating strong binding affinities to the GLP-1 receptor, with 1 exhibiting the highest XP score of −13.835. The MM-BBSA dG Bind values, indicative of binding affinity and measured in kcal/mol for these hits, ranged from −84.69 to −66.07, showing a significant correlation with the XP scores and further implying strong interactions between the ligands and the receptor. The Shape Similarity value reflects the geometric similarity of the hits compared to a reference ligand, with hits falling in the intermediate similarity range of 0.305–0.346 compared to the control compound (the co-crystallized agonist PF-06882961) (Shape Similarity = 1.000). In the Marine database, the mean XP scores for the top 10 hit compounds were between −12.80 and −10.190 kcal/mol. The best among them is 11, which achieved the highest binding affinity (−12.82 kcal/mol). The binding energies—MM-BBSA dG_Bind values for those compounds are between −100.43 and −52.04 kcal/mol, from which compound 16 exhibited the most favorable chemical affinity of −100.43 kcal/mol. As for the comparisons of Shape Similarity of Marine hits from 0.302 to 0.375, different compounds fit moderate to high degrees of similarity to the control compound. It should be noted that only one compound from the Marine database, 15, obtained a Shape Similarity score of 0.375, which further underpins its potential as a promising hit. The results confirm the existence of compounds within the COCONUT and Marine databases that possess strong binding affinities and some promising shape characteristics to the reference ligand, making them suitable for further examination as non-peptide GLP-1RAs. Favorable binding-based 2D and 3D docking interactions with key amino acid residues involved LYS-197, TRP-203, and PHE-381, which proved essential for the high-affinity interactions observed with the GLP-1R binding pocket (Figure 3). These results imply that the screened compounds are promising hits for targeting the GLP-1 receptor, enabling a more potent and selective interaction.

Analysis of the binding interactions of the identified ligands was based on identifying their interactions with the key residues identified in the crystal structure as crucial for binding the GLP-1 peptide, namely, LYS-197, TRP-203, and PHE-381. Common additional interactions were also searched to identify potential binding poses and important residues that might drive future drug design and development.

Strong π–cation interactions were noted for hit **1** with the pyridine nitrogen and TRP-203 and PHE-381. The indole ring further anchored the compound through a π–π interaction with TRP-33. An additional H bond was established between the acetate side chain and GLN-221.

Hit **5** was found to bind the active site with a similar binding pose to hit **1**. The pyridine nitrogen interacted through π–cation interactions with TRP-203 and PHE-381. The polycyclic aromatic system established several π–π interactions with TRP-33, and the dioxolane oxygen interacted with an H bond as acceptor with GLN-221. The same interactions were completely observed between hit **9** and the same previously mentioned residues. Hit **11**, however, lacked the interaction with PHE-381 while keeping the same interaction profile with TRP-203 and GLN-221. The missed interaction was compensated for by additional H bonds with SER-31 and LEU-32. On the other hand, hit **13** lacked the interaction with PHE-381 without additional bonding (Figure 3).

In summary, the binding profile of the top hits exhibited consistent binding with the key residues TRP-203 and GLN-221 through π–cation or π–π interactions and H bond as acceptor, respectively. The binding analysis also identified TRP-33 as an important binding residue that would act as a gatekeeper at the entrance of the binding site and that is preferably bound through a π–π interaction with a planar aromatic system. Moreover, the high values of the docking scores (−13) and binding energy calculations (−85) validated the significance of the drawn binding pattern and residues.

### 2.3. MD Simulations and Binding Free Energy Calculations for MD Frames

**Figure 4:**
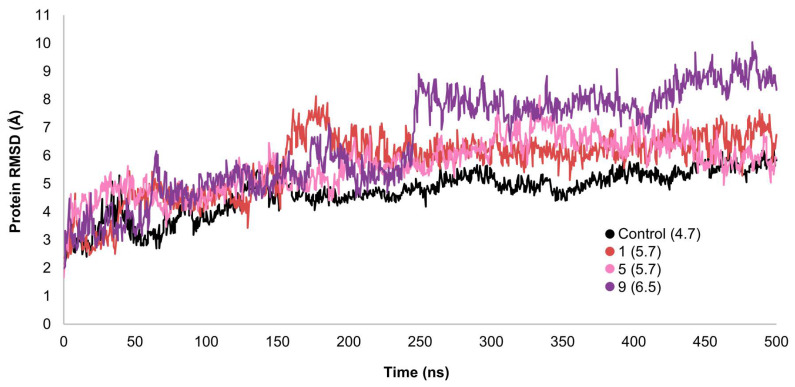
RMSD analysis for protein Cα atoms in different complexes. Average RMSD values are shown in parentheses.

In mutagenesis studies, the activity of the non-peptide control compound was found to be reduced by alanine mutations of many residues, including ARG-380, GLU-373, PHE-385, LYS-197, TRP-33, PHE-230, LEU-141, TRP-203, PHE-381, LEU-384, and ARG-190 [9]. With this knowledge, we have used MD simulations over a period of 100 and 500 nanoseconds to evaluate the stability of ligand-target complexes, the binding poses from the docking calculations, and the interaction pattern between each ligand with key GLP-1R amino acids. The studies were performed for top select candidates from virtual screening, namely compounds **1**, **5**, and **9**, and for the control compound as a reference. To assess the stability of the protein, we evaluated the RMSD values of Cα atoms (Figure 4).

**Figure 5:**
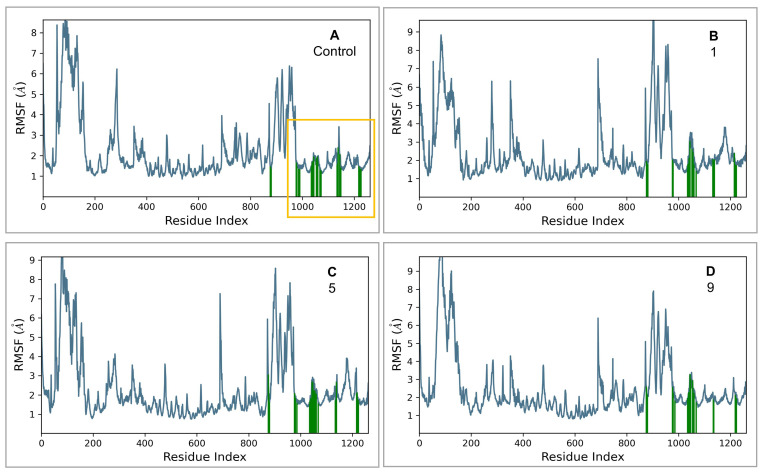
RMSF analysis for C*α* atoms in different complexes. The TM region is indicated in the yellow box. Residues that interact with each ligand are shown as green vertical bars. (**A**) control, (**B**) compound **1**, (**C**) compound **5**, and (**D**) compound **9**.

The proteins in the complexes of the control and compounds **1** and **5** stabilized at around 200 ns, while the protein in the **9**-GLP-1R complex stabilized at around 250 ns. The proteins in all complexes remained stable for the rest of the simulation time with no significant structural deviations, indicating that they had reached equilibrium. Fluctuations from average Cα-atom RMSD values were within an acceptable range of 1–3.5 Å, which is expected since the GLP-1R protein is a large, multi-domain, G-protein coupled structure. The average protein Cα atoms RMSD values were in the range of 4.7–6.5 Å. RMSF analysis shows that the ligand binding site in the TM region remains stable throughout the simulation for all the complexes (Figure 5).

**Figure 6:**
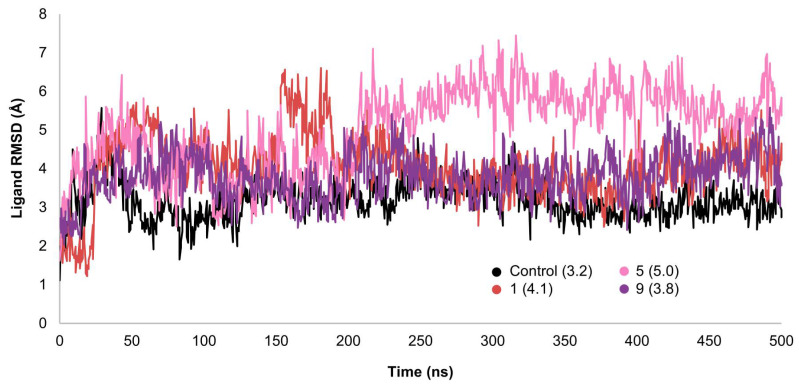
RMSD analysis for ligand heavy atoms in different complexes. Average RMSD values are shown between parentheses.

To assess ligand stability, RMSD values for ligand heavy atoms were measured with respect to the protein (Figure 6). None of the average ligand heavy-atom RMSD values is significantly larger than the average protein Cα atom RMSD value. This finding indicates that compounds **1**, **5**, **9**, and the control were all bound efficiently within the binding site, which was also confirmed visually (Supplementary Materials Figures S1–S3, residues that interact for 45% or more of the simulation time are shown). Based on RMSD analysis and visual inspection, compound **5** seems to have changed its orientation after 200 ns has passed, but it remains stable. Similar to the control, compound **9** remains stable throughout the simulation time. Compound **1** shows some fluctuations within the binding side until the pyridoindole group is in an optimum position to form H-bonds with GLN-221, and the alcohol group is in an optimum position to form H-bonds with THR-298 and ARG-299. Compound **1** then binds stably to these residues for the rest of the simulation time. Except for compound **1**, all ligands had no major structural deviations from their starting docking conformations. Average RMSD values for ligand heavy atoms ranged from 3.2–5 Å. Compounds **9** and **1** had the closest average RMSD values to the control, while compound **5** had the highest RMSD value.

**Figure 7:**
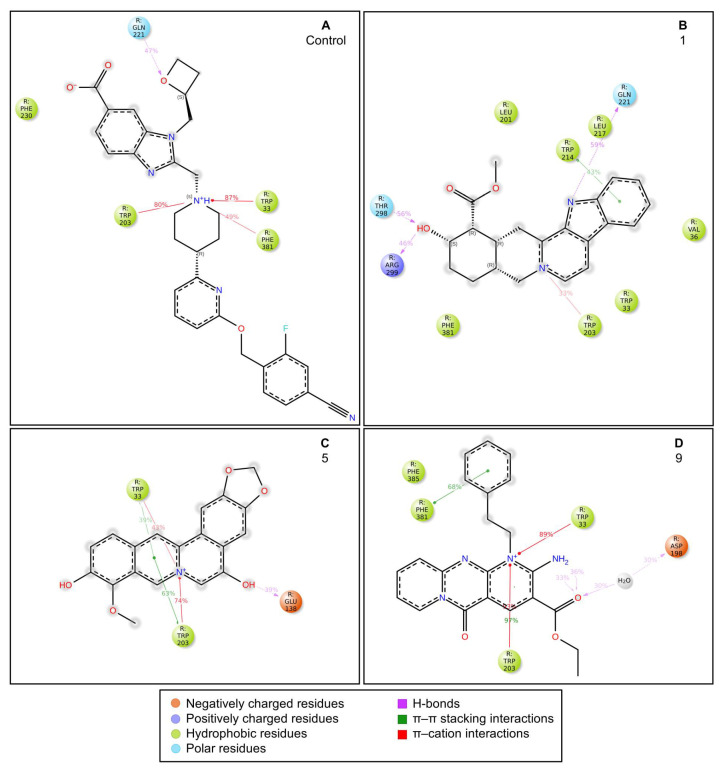
Interactions between the ligands and residues in the binding site of GLP-1R during MD simulations. (**A**) control, (**B**) compound **1**, (**C**) compound **5**, and (**D**) compound **9**.

- Control-GLP-1R complex
  The control compound forms a hydrogen bond with GLN-221. More importantly, it forms strong and stable π–cation interactions with three key amino acids, TRP-33, TRP-203, and PHE-381, for 87%, 80%, and 49% of the simulation time, respectively (Figure 7A). These key interactions stabilize the complex and contribute to the known agonist activity of the control compound. The average binding free energy for the control-GLP-1R complex was −97.30 kcal/mol.
- Compound **1**-GLP-1R complex

Compound **1** forms H-bonds with GLN-221, THR-298, and ARG-299 (Figure 7B), and the former two can stabilize the complex since they last for more than half the simulation time. It also forms a π–π stacking interaction with TRP-214 and a π–cation interaction with TRP-203. Although TRP-203 is important for the activity of non-peptide GLP-1R agonists, the interaction of this residue with compound **1** is not strong. The average binding free energy for the compound **1**-GLP-1R complex was −97.87 kcal/mol, similar to the control ligand. This could indicate that compound **1** is a good binder.

- Compound **5**-GLP-1R complex

Compound **5** forms a H-bond with GLU-138 (Figure 7C). It also forms π–π stacking interactions and π–cation interactions with two key amino acids, TRP-33 and TRP-203, with the latter lasting for more than half the simulation time. Such strong interactions with TRP-203 can stabilize the complex and contribute to compound activity, potentially as a GLP-1R agonist. The average binding free energy for the compound **5**-GLP-1R complex was −92.50 kcal/mol, a value close to that of the control ligand. These findings indicate that compound **5** may be a potential GLP-1R agonist.

- Compound **9**-GLP-1R complex

Compound **9** forms a H-bond with ASP-198 through a water bridge (Figure 7D). It also forms π–π stacking interactions with important amino acids TRP-203 and PHE-381 for more than half the simulation time. Stable π–cation interactions are also observed between compound **9** and the key amino acids TRP-33 and TRP-203, lasting for 89% and 93% of the simulation time, respectively. The average binding free energy for the compound **9**-GLP-1R complex was −102.78 kcal/mol, the lowest value among all four complexes. Compound **9** seems the most promising as a GLP-1R agonist.

The average MM-GBSA Δ*G*_bind_ values obtained from the MD simulation frames of the control and the three compounds were low, comparable, and indicative of strong binding. The affinity ranking of compounds based on MM-GBSA Δ*G*_bind_ values often correlates with the ranking observed in experimental settings. Accordingly, the binding affinity of the ligands can be predictively ranked as **9** (−102.78 kcal/mol) > **1** (−97.87 kcal/mol) > control (−97.30 kcal/mol) > **5** (−92.50 kcal/mol). Compound **9** outperformed compounds **1**, **5**, and the control in terms of predicted binding affinity.

Results from the MD simulations and the binding free energy calculations indicate that compound **9** is the most promising since it demonstrates the highest predicted affinity. For most of the simulation time, this compound maintains stable interactions with three key amino acids that are important for non-peptide agonist activity. Its binding profile in terms of energy, interactions, and stability within the binding site is similar to, and sometimes better than, that of the control, which further supports its potential as a promising non-peptide GLP-1R agonist. On the other hand, compound **1** has favorable energetics and seems to strongly bind to GLP-1R through different amino acids than the other three compounds. This warrants further investigation into its activity to determine whether such binding behavior will also result in GLP-1R activation. Compounds **1** and **9** have comparable ligand heavy-atom average RMSD values to the control and remain stable in the binding site. Compound **5** maintains interactions with one important amino acid for more than half the simulation time and has a favorable binding free energy. All three hits interact with at least one residue for more than 50% of the simulation time, show protein stability, and exhibit minimum structural fluctuations for at least half of the simulation time.

### 2.4. Literature Chemical Scaffolds Analysis

Despite not having direct experimental or clinical evidence concerning the exact molecules, a complete review of specific compounds from the COCONUT and CMNPD (marine) databases reveals a diverse array of structural classes with potential anti-diabetic properties (Table 4 and Table 5). Among the compounds originating from coconut, CNP0593098.1 (3,4,5,6-tetradehydroyohimbine) stands out. No research has directly addressed this derivative, but anti-diabetic activity has been demonstrated for its parent compound, yohimbine, in animal models via α2-adrenoceptor antagonism, resulting in increased insulin secretion and improved glucose tolerance. Neither this derivative nor other derivatives of yohimbine have, however, been able to show GLP-1 agonist activity.

Other compounds, as found from COCONUT, include CNP0402650.0 and CNP0311770.0 (chromen derivatives). These belong to structural classes with an established reputation for antioxidant activities, inhibitory effects on carbohydrate-hydrolyzing enzymes (like α-glucosidase), and insulin-sensitizing effects. Despite this, no studies have researched these specific molecules for these functions. Other compounds from coconut, such as CNP0542406.3, CNP0510864.0, and CNP0294111.0, are also characterized by their structural cores of indole, pyridine, and benzofuran, which are recurrent in anti-diabetic studies, mainly for their oxidative stress reduction, insulin sensitizing, and pancreatic β-cell protecting effects; once again, no literature records exist for these particular compounds.

Continuing in the same vein, marine-derived compounds cataloged under CMNPD appear to harbor equally enticing avenues. Some of the quinazoline alkaloids such as CMNPD5314 (fumiquinazoline B) and CMNPD27343 (versiquinazoline B) resemble structurally known compounds that inhibit α-glucosidase and protein tyrosine phosphatase 1B (PTP1B), two well-studied anti-diabetic targets. Other marine substances, comprising CMNPD2041 (waixenicin A), CMNPD24592 and CMNPD24593 (speradines D and E), and CMNPD26002 (a gliotoxin derivative), are diterpenes and alkaloids that are known, at least in structurally related forms, for anti-inflammatory and metabolic regulating actions, including PTP1B inhibition and AMPK activation.

Marine-derived epipolythiodioxopiperazines, in addition, activate the Nrf2/HO-1 pathway and thus would confer cytoprotective and antioxidant effects that preserve β-cell function. Even though there are no direct studies regarding anti-diabetic or GLP-1 activities for specific compounds such as CMNPD27661, CMNPD4014, CMNPD9270, and CMNPD22795, the structural classes to which they belong, including polyketides, prostaglandin derivatives, and benzofurans, are increasingly associated with redox modulation, enzyme inhibition, and generalized metabolic benefits.

In summary, compounds from both coconut and marine natural product databases present strong potential as anti-diabetic agents from the standpoints of structural features, mechanistic plausibility, and class-based pharmacological evidence. Compounds from coconut are notably important for insulinotropic and β-cell-protective effects, while those marine ones would provide additional scaffolds for enzyme and redox pathways involved in glucose homeostasis. Although bioactivity data on these particular molecules are absent, the confluence of evidence available for their structurally related counterparts renders them promising agents in multi-target anti-diabetic drug discovery and clearly worthy of intensive evaluation.

The literature-supported findings affirm the pharmacophore hypotheses and in silico prediction proposed in the current investigation. They promote the contention that natural product scaffolds, selected via rational filters combined with docking and shape-similarity methods, constitute a rich source of non-peptidic GLP-1 receptor modulators.

### 2.5. ADMET and Drug-Likeness

In Table 6, the water solubility of all compounds ranges from −3.851 to −2.791 log mol/L, with **3** exhibiting the highest solubility and compound 6 showing the lowest solubility. Despite this difference, most compounds have moderate to low solubility, with **5** below average. With respect to the Caco-2 permeability scale, **1**, **2**, **5**, **6**, **8**, and **9** can be stated to have good membrane permeability (>0.5 log Papp), allowing easy passage across the intestinal epithelial barriers; while **3**, **4**, **7**, and **10**, get below this threshold, showing less permeability. Intestinal absorption from the human GI tract is generally high, above 95% for compounds **1**, **2**, **5**, **6**, **8**, and **9**, implying good oral bioavailability. On the contrary, **3** is poorly absorbed (3.62% absorption), with subpar values for **4** and **10** (48%). Nearly all the candidates are P-glycoprotein substrates, except for **3** and **7**, which probably increase retention inside the cell.

On the permeability of the blood–brain barrier (BBB), all compounds show a log BB value under the +0.3 threshold, thereby implying low or just moderate penetration into the CNS. Compounds **1**, **2**, and **8**, albeit close to this threshold, are considered moderate. CNS Permeability (log PS) values are uniformly low, as all compounds apart from compound **8** (−1.894) are below the −2.0 mark, confirming little CNS exposure and so little risk of CNS-associated side effects.

Compounds **1**, **2**, **6**, and **8** are substrates of CYP3A4. Compounds **3** and **4** are substrates of CYP2D6, which may impede their metabolic stability. Regarding CYP450 inhibitory activity, the scenario changes since **1**, **2**, **4**, **5**, **6**, and **8** inhibit at least one isoform, interfering with the metabolism of some co-administered drugs. Compound **4** inhibits three CYP enzymes (i.e., CYP1A2, CYP2C9, and CYP3A4) and can, therefore, be considered at greater risk for metabolic interaction. Compounds **3**, **7**, and **10** show little or no CYP inhibition and therefore seem to possess a clean metabolic profile.

Total clearance is from 0.099 to 1.189 log mL/min/kg. A greater clearance above 1 is for **1**, **2**, **5**, and **8**, which means that they are quickly eliminated systemically. Compound **7** has a very low clearance value and therefore remains exposed longer. Only compounds **5** and **8** are substrates of renal OCT2, suggesting that renal excretion may occur, a factor that should be kept in mind when considering dosage for patients with renal impairment.

Toxicological profiling is suspected to be a risk for several candidates. Compounds **1**, **2**, and **8** are AMES-positive, which points toward mutagenicity. The maximum tolerated dose varies among compounds **3**, **4**, **6**, **7**, and **10**, and is generally high. Hepatotoxicity seems to be the leading cause for concern, observed in all except compounds **5** and **7**, thus supporting the presumption that liver safety is a top priority. It is interesting to note that no compounds are considered to inhibit hERG I channel, thus leaving cardiotoxicity out of consideration.

Most of the compounds (**1**, **2**, **5**, **6**, and **8**) exhibit appreciable absorption; however, compound **3** is poorly soluble and absorbed, and thus requires structural modification. None of the compounds are presumed to cross the BBB or to promote significant CNS penetration. Compounds **3**, **7**, and **10** exhibit minimal CYP inhibition, whereas compounds 4 and 6 demonstrate high metabolic liability. Most compounds were cleared efficiently, with compound **7** being an outlier. In terms of toxicity, compound **5** exhibits a more favorable profile, being free from AMES toxicity and hepatotoxicity. These profiles are used to lay the groundwork for prioritizing experimental validation.

The physicochemical and drug-likeness profiling of the tested compounds revealed overall favorable properties with respect to oral drug development guidelines (Table 7). Most compounds exhibited molecular weights below the 500 Da threshold, except for the control (543.59 Da), aligning with Lipinski’s rule of five. LogP values ranged from 0.68 to 3.22, indicating moderate lipophilicity, and most compounds had acceptable numbers of hydrogen bond donors and acceptors. Polar surface area (TPSA) was within reasonable values (mainly < 140 Å^2^), which are suitable for good membrane permeability, except for **3** (152.08 Å^2^). Most molecules met more than one drug-likeness filter (Ghose, Veber, Egan, and Muegge), but **3** always diverged and were not capable of crossing all filters and were more flexible and polar. PAINS and Brenk alerts were low across the dataset, suggesting a low risk of assay interference or structure risk. Synthetic accessibility scores ranged from 2.68 to 4.75, which translates to moderate to excellent synthetic viability. The data in total give support to the validity of these molecules as potential drug-like hits, with **3** and the control when it comes to polarity and complexity.

## 3. Materials and Methods

### 3.1. Materials and Software

For our computational studies, we utilized the Schrödinger’s Maestro molecular modeling software (Versions 2024-4 and 2025-1) [32,67,68]. Our computational work was conducted on a desktop workstation equipped with an Intel^®^ Core™ i7-10700F Processor (Intel, Santa Clara, CA, USA), running the Linux Ubuntu 22.10 Operating System, and featuring an RTX 5000 graphics card (RDIA 12990-iau-2023-iau-R-3-1-HW: P.O. 6947 License key: 03cb87b8-723c-4fec-9b8c-8a58137d7a76).

### 3.2. Database Preparation

We obtained a remarkable comprehensive natural products (NPs) database that contains 695,133 NPs from the Coconut website https://coconut.naturalproducts.net/ (accessed on 15 November 2024) [35]. In addition, we downloaded over 46,000 compounds from the Comprehensive Marine Natural Products database (CMNPD), which was retrieved from the online server (https://www.cmnpd.org/, accessed on 18 November 2024) [36].

The collected structures were filtered using Schrödinger’s Canvas based on their physicochemical descriptors, including MW, logP, number of rings, total heavy atoms count, overall charge, number of H-donors, number of H-acceptors, number of rotatable bonds, total charge, and QED score. The final database filtration ensured compliance with the rule of five [69] and maintained a natural product-like (NPL) score < 2 [70]. The NPL score, introduced by Ertl et al. is a numerical measure that quantifies how much a chemical structure resembles a natural product compared to a synthetic molecule. The NPL score ranges between −5 (if the compound is more similar to a synthetic compound) and 5 (if the compound is more similar to a natural product). A score of 0 or higher is often used to distinguish between natural and synthetic molecules. The higher the score, the more similar the molecule is to natural product structures. It is used to prioritize molecules for screening in drug discovery programs [71]. Given the big number of compounds screened in our research, we used a cut-off of 2 for NPL scores to exclude compounds that are more natural product like and would have higher probability of being orally inactive.

### 3.3. Shape Screening

Our investigation was carried out using Schrödinger’s Maestro’s form screening tool. We started by adding a default RMSD of 0.30 Å, specifically for non-hydrogen atoms, to the filtered database in order to decrease its energy using the Optimized Potentials for Liquid Simulations 3 (OPLS3) force field. Following this optimization, shape screening was carried out with the crystal ligand structure serving as a guide. We used a number of different pharmacophore types in conjunction with a volume-scoring algorithm to precisely evaluate the compounds. We ranked the compounds according to their shape similarity score, setting a limit of 0.3 to further refine our virtual screening database. The evaluation was based on pharmacophore features [72].

### 3.4. Crystal Structures

**Figure 8:**
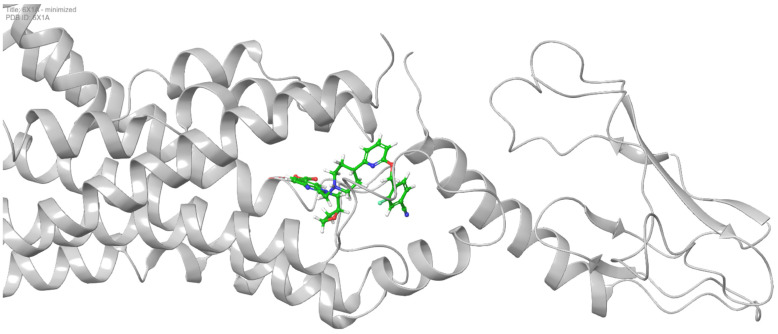
The crystal structure of the orthosteric GLP-1 receptor (PDB ID: 6X1A) is depicted as a cartoon model, with the non-peptide agonist PF-06882961 bound to the receptor. The PF-06882961 molecule is shown as sticks, with carbon atoms colored in green.

The crystal structure of GLP1 in complex with the non-peptide agonist PF-06882961 (PDB ID: 6X1A) was obtained from the Research Collaboratory for Structural Bioinformatics (RCSB) Protein Data Bank (PDB) (Figure 8) [73].

### 3.5. Protein Preparation

This protein structure was prepared for docking using Maestro’s Protein Preparation Workflow. To fine-tune the ionization states, the preparation and minimization process were carried out at a pH 7.4. We added polar hydrogens and eliminated extraneous water molecules from the structures. Using the OPLS3 force field to optimize the receptors was the last stage. Under the OPLS3 force field, the ligand-protein complexes were optimized and minimized using a preset RMSD value of 0.30 for non-hydrogen atoms [74]. Then, receptor grids with a radius of 1.00 A van der waals (vdW) were made using the center of the bound ligand. In this procedure, a vdW radius was applied and the threshold for partial charges was set at 0.25 [75]. The binding sites were contained within a grid box of 20 Å using default parameters and without any constraints

### 3.6. Ligand Library Preparation

The LigPrep tool (Versions 2024-4), integrated into the virtual screening workflow of Maestro, was used to prepare the filtered ligands. The three-dimensional structures of the ligands were created by the addition of hydrogen atoms, followed by the generation of the most probable ionization states at pH 7 ± 2 using Epik. The geometry of the ligands was then optimized with the OPLS3 force field, which included tautomer generation, desalting, and producing a maximum of 32 isomers per ligand [67,76]. The resulting conformations served as the starting input structures for the virtual screening workflow.

### 3.7. Validation of Molecular Docking

The molecular docking protocols were evaluated in order to confirm Maestro Glide’s accuracy in predicting the ligand’s docking poses for the protein under study [77,78,79]. The library screening criteria were used to re-dock the cognate ligand into the GLP1 receptor.

To validate the robustness of the molecular docking protocol, a cross-docking experiment was conducted on GLP-1R. Three high-resolution crystal structures of GLP-1R in complex with different small-molecule ligands were retrieved from the Protein Data Bank (PDB): 6X1A, 6X19, and 6ORV. These structures represent slightly different conformations of the receptor–ligand binding domain. PF-06882961 was extracted from its native complex (PDB: 6X1A) and docked into all three receptor grids using the Glide Extra Precision (XP) docking protocol in Maestro. The results were calculated using the Ligand RMSD tool and GlideScore (Versions 2024-4).

### 3.8. Virtual Screening Workflow

**Figure 9:**
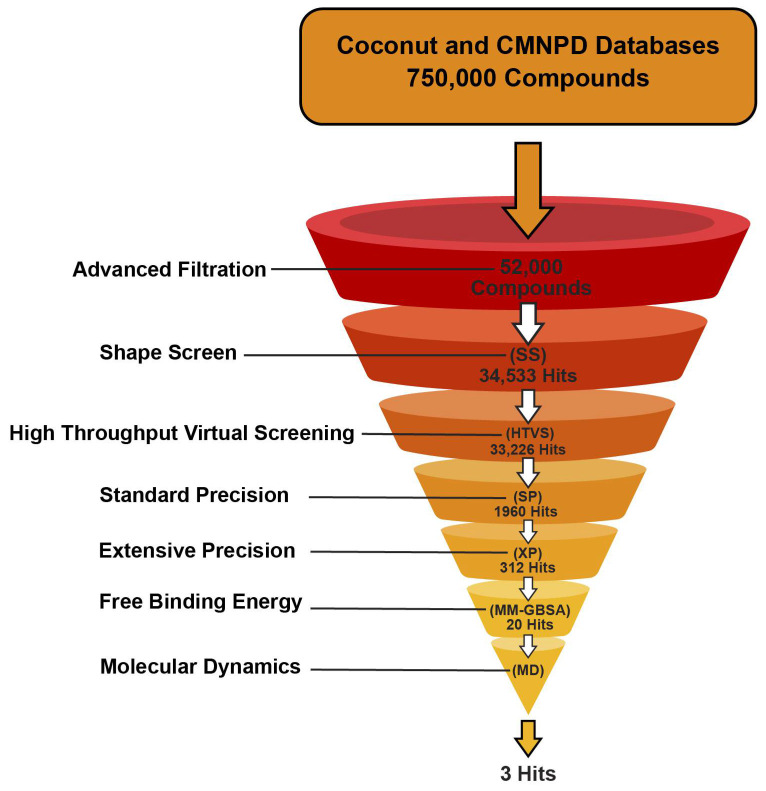
Overview of the non-peptide GLP-1RA in silico identification workflow used in this study.

Three distinct methods were employed to structure the virtual screening docking workflow: High-Throughput Virtual Screening (HTVS), Standard Precision (SP), and Extra Precision (EP). Therefore, flexible docking was utilized in conjunction with post-docking minimization for each approach, where each compound generated three poses and retained up to 10% of the highest-scoring compounds; this filtration criterion is acceptable when screening large databases [32]. A more rigorous hit identification stage was then applied to the initial filter through XP docking, MM-GBSA calculation, and lastly 500 ns MD simulations for the final hits. During the docking procedure, no additional filters or constraints were applied. The actual parameters included a vdw radius scaling factor of 0.80 and a partial charge cut-off of 0.15. The ligands and hit molecules were ranked using the XP score. For binding free energy calculations in molecular mechanics-generalized Born surface area (MM-GBSA), the virtual screening pipeline estimated the binding affinity of top-scoring hits using the Prime MM-GBSA module (Figure 9).

### 3.9. Molecular Dynamics (MD) Simulations

Molecular dynamics (MD) simulations were conducted for compounds **1**, **5**, **9**, and the control compound, using the Desmond Module in the Schrödinger suite [80,81,82]. Desmond’s system builder tool was used to prepare the protein–ligand complexes for MD simulations. Since GLP-1R is a transmembrane (TM) protein and our ligands mainly bind in the TM region, a 1-palmitoyl-2-oleoyl-sn-glycero-3-phosphocholine (POPC) lipid bilayer membrane was added at 300 K [83]. The simple point charge (SPC) water model was used for an orthorhombic box with dimensions of 10 Å × 10 Å × 10 Å [84]. A 20 Å distance from the ligand covered the region for the exclusion of ion and salt placement. The number of required counter ions was calculated and subsequently added to neutralize the system. Sodium and chloride ions were used to maintain a 0.15 M salt concentration, and the OPLS5 force field was selected for running the simulations [85]. The environmental conditions included a temperature of 300 K, a pressure of 1 bar, and the NP γT ensemble were set [86,87]. The membrane model system was relaxed before running 100–500 ns simulations. Coordinates were recorded every 500 ps, and 1000 frames were generated for the trajectory. The temperature was controlled using the Nose–Hoover chain coupling scheme with a 1 ps coupling constant. The pressure was controlled using the Martyna–Tobias–Klein coupling scheme with a 2 ps coupling constant. A time step of 2 fs was selected for RESPA (Reference System Propagator Algorithm), and a 9 Å cutoff radius was set for Coulombic short-range interactions [87]. Desmond’s Simulation Interaction Diagram tool was used to analyze data generated from the MD simulations [88]. The RMSD values were evaluated to determine how stable the ligand–protein complexes are, and root-mean square fluctuation (RMSF) was used to assess individual residue fluctuations [89].

### 3.10. Binding Free Energy Calculations for MD Frames

The binding free energy was calculated for frames generated from MD simulations for compounds **1**, **5**, **9**, and the control. Schrödinger’s MM-GBSA continuum solvation method and the thermal_mmgbsa script were utilized [90]. A step size of five was used, and average ΔGbind values were reported. These calculations provide more thorough and accurate insights into the binding energetics for these compounds and can help rank them based on predicted affinities [91,92].

### 3.11. ADMET Profiling

To predict the ADMET (absorption, distribution, metabolism, excretion, and toxicity) properties as well as drug-likeness characteristics for the selected putative agonists used as modeling input, we utilized the pkCSM web server (http://biosig.unimelb.edu.au/pkcsm/prediction (accessed on 13 December 2024)) [92]. Eight molecular descriptors were generated based on the ADMET descriptors identified in the potential hits. Additionally, Swiss ADME (www.swissadme.ch/ (accessed on 13 December 2024)) was employed for the in silico calculations of physicochemical parameters, medicinal chemistry-like properties, and drug-likeness attributes [93,94].

## 4. Conclusions

More than 700,000 compounds from natural sources, obtained from the Coconut and Marine Natural Products (CMNPD) libraries, were screened for agonist activity at the orthosteric site of GLP-1R to identify novel non-peptide compounds with better clinical utility. A filtration protocol combining both receptor-based and ligand-based approaches was utilized, including shape screening, precision docking, binding energy (MM-GBSA) calculations, and 500 ns molecular dynamics simulations. Pharmacokinetics prediction was also performed on the hit compounds to establish their drug-likeness potential and clinical applicability. Twenty final hits were identified from both databases, with some hits, including **1**, **6**, **7**, and **10**, having previously reported antidiabetic activity but no evidence of GLP-1 activity. The most active hits, **1**, **5**, and **9**, showed an excellent binding profile, with key residues and binding stability as indicated by the binding scores, RMSF, and RMSD values, respectively. These results paved the way for further experimental studies to confirm the GLP-1-mediated antidiabetic activity of these hits. These results also substantiated the primary identification of other hits and positioned them as novel non-peptide structural scaffolds for GLP-1 orthosteric agonist activity with subsequent antidiabetic effect. The predicted ADMET profiling was acceptable, particularly in terms of drug-likeness and bioavailability. The current study identified new lead compounds for GLP-1R orthosteric agonist activity that, on the one hand, represent novel non-peptide scaffolds for optimization and preclinical development, and on the other hand, propose GLP-1R-mediated activity for those hits with known antidiabetic activity, warranting further experimental testing.

## Figures and Tables

**Table 1:** Cross-docking results for PF-06882961 into three GLP-1R crystal structures.

Ligand	Receptor (PDB)	RMSD (Å)	GlideScore
PF-06882961	6X1A (Native)	2.03	−12.040
	6X19	1.15	−9.540
	6ORV	1.19	−8.885

**Table 2:** Structure, XP score, MM-GBSA, and shape similarity of the top 10 hits from the COCONUT database with the GLP-1R binding site (PDB ID: 6X1A).

Hit No. *	Structure	ID	XP Score	MM-BBSA dG Bind	Shape Similarity *
**1**	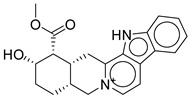	CNP0593098.1	−13.835	−84.69	0.346
**2**	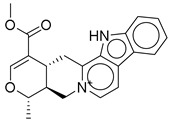	CNP0542406.3	−13.657	−83.94	0.315
**3**	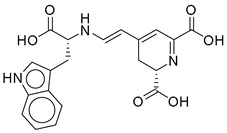	CNP0510864.0	−13.513	−82.51	0.305
**4**	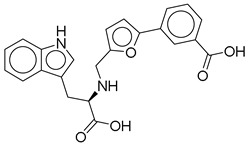	CNP0294111.0	−13.506	−84.61	0.344
**5**	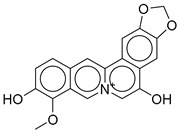	CNP0513629.0	−13.114	−76.49	0.308
**6**	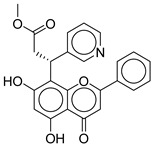	CNP0402650.0	−13.003	−86.43	0.325
**7**	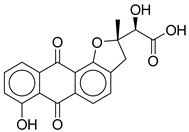	CNP0449126.4	−12.954	−66.07	0.320
**8**	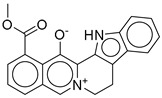	CNP0576495.0	−12.770	−76.87	0.312
**9**	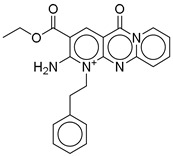	CNP0510059.0	−12.563	−84.80	0.339
**10**	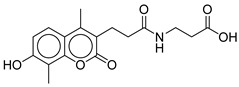	CNP0311770.0	−12.532	−85.80	0.310
**Control**	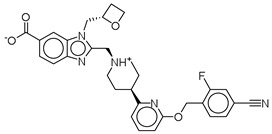		−12.040	−116.06	1.000

* Shape similarity of the hits and control. Similarity ranges: 0.5–1 (High), ≥0.3–0.49 (Intermediate), <0.3 (Low). Cutoff score ≥ 0.3.

**Table 3:** Structure, XP score, MM-GBSA, and shape similarity of the top 10 hits from the Marine database with the GLP-1R binding site (PDB ID: 6X1A).

Hit No. *	Structure	ID	XP Score	MM-BBSA dG Bind	Shape Similarity *
**11**	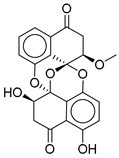	CMNPD27661	−12.806	−71.68	0.303
**12**	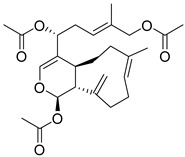	CMNPD2041	−11.609	−96.80	0.327
**13**	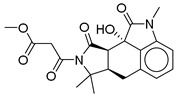	CMNPD24592	−11.575	−74.09	0.315
**14**	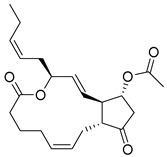	CMNPD4014	−11.495	−95.02	0.302
**15**	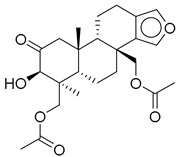	CMNPD9270	−11.427	−97.33	0.375
**16**	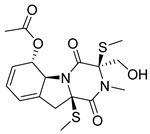	CMNPD26002	−11.116	−100.43	0.320
**17**	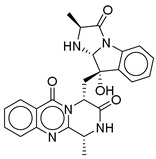	CMNPD5314	−10.805	−75.01	0.343
**18**	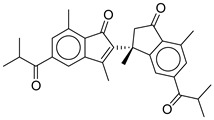	CMNPD22795	−10.798	−88.67	0.363
**19**	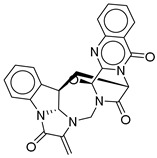	CMNPD27343	−10.372	−61.54	0.311
**20**	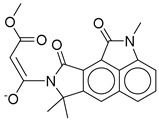	CMNPD24593	−10.190	−52.04	0.352
**Control**	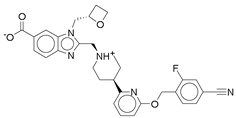		−12.040	−116.06	1.000

* Shape similarity of the hits and control. Similarity ranges: 0.5–1 (High), ≥0.3–0.49 (Intermediate), <0.3 (Low). Cutoff score ≥ 0.3.

**Table 4:** Potential antidiabetic candidate molecules from the COCONUT database.

COCONUT ID	Compound Name/Description	Anti-Diabetic/GLP-1 Literature Evidence (2015–2025) [41,42,43,44,45,46,47,48,49,50,51]
CNP0593098.1	3,4,5,6-Tetradehydroyohimbine	Yohimbine (parent compound) has demonstrated antidiabetic effects in STZ-induced diabetic rats. It acts as an alpha-2 adrenoceptor antagonist, improving glucose tolerance, lowering blood glucose, and improving lipid profiles. The mechanism is thought to involve increased insulin secretion by blocking inhibitory catecholaminergic tone in pancreatic islets. No direct evidence for GLP-1 agonism or for the specific dehydro-derivative.
CNP0542406.3	(15S)-19-(methoxycarbonyl)-16-methyl-17-oxa-3,13λ^5^-diazapentacyclo [11.8.0.0^2,10^.0^4,9^.0^15,20^]henicosa-1(13),2(10),4,6,8,11,18-heptaen-13-ylium	No direct literature for this structure
CNP0510864.0	4-[2-[[1-carboxy-2-(1H-indol-3-yl)ethyl]amino]ethenyl]-2,3-dihydropyridine-2,6-dicarboxylic acid	No direct literature for this structure
CNP0294111.0	3-[5-[[[1-carboxy-2-(1H-indol-3-yl)ethyl]amino]methyl]furan-2-yl]benzoic acid	No direct studies.
CNP0513629.0	16-methoxy-5,7-dioxa-13-azoniapentacyclo [11.8.0.0^2,10^.0^4,8^.0^15,20^]henicosa-1,3,8,10,12,14,16,18,20-nonaene-11,17-diol	No direct anti-diabetic or GLP-1-related studies for this structure.
CNP0402650.0	methyl 3-(5,7-dihydroxy-4-oxo-2-phenyl-4H-chromen-8-yl)-3-(pyridin-3-yl)propanoate	Chromen derivatives and flavonoids are widely studied for anti-diabetic effects, often through antioxidant and insulin-sensitizing mechanisms, but no direct evidence
CNP0449126.4	(2S)-2-hydroxy-2-[(2S)-7-hydroxy-2-methyl-6,11-dioxo-3H-naphtho[2,3-g]benzofuran-2-yl]acetic acid	Benzofuran derivatives have shown anti-diabetic effects but no direct study for this compound.
CNP0576495.0	19-methoxycarbonyl-3-aza-13-azoniapentacyclo[11.8.0.0^2,10^.0^4,9^.0^15,20^]henicosa-1(13),2(10),4,6,8,14,16,18,20-nonaen-21-olate	No direct literature for anti-diabetic or GLP-1 effects.
CNP0510059.0	ethyl 6-amino-2-oxo-7-(2-phenylethyl)-1,9-diaza-7-azoniatricyclo[8.4.0.0^3,8^]tetradeca-3(8),4,6,9,11,13-hexaene-5-carboxylate	No direct literature for anti-diabetic or GLP-1 effects.
CNP0311770.0	STL522422; 3-[3-(7-hydroxy-4,8-dimethyl-2-oxo-chromen-3-yl)propanoylamino]propanoic acid	Chromen (coumarin) derivatives show anti-diabetic effects (antioxidant, insulin-sensitizing)

**Table 5:** Antidiabetic Relevance of Selected CMNPD Compounds: Structural Classes, Mechanistic Insights.

CMNPD ID	Compound Name	Structural Class/Source	Direct Anti-Diabetic/GLP-1 Evidence [52,53,54,55,56,57,58,59,60,61,62,63,64,65,66]	Mechanistic/Structural Notes
CMNPD27661	preussomerin M	Polyketide (fungal)	No direct anti-diabetic or GLP-1 literature was found for this compound.	Polyketide-type marine metabolites are being explored as anti-diabetic
CMNPD2041	waixenicin A	Diterpene (soft coral)	No direct anti-diabetic or GLP-1 literature found for this compound.	Marine diterpenes show anti-inflammatory and metabolic regulatory effects.
CMNPD24592	speradine D	Alkaloid (marine fungus)	No direct studies for this structure; marine alkaloids are reported to enhance glucose uptake, insulin sensitivity, and AMPK activity.	Alkaloids from marine sources are highlighted as promising anti-diabetic agents via AMPK/GLUT4 pathways.
CMNPD4014	PGE3-1,15-lactone-11-acetate	Prostaglandin derivative	No direct anti-diabetic or GLP-1 evidence; some marine prostaglandins have anti-inflammatory and metabolic effects.	Prostaglandin derivatives may indirectly affect glucose metabolism via inflammation modulation.
CMNPD9270	speradine E	Benzofuran derivative	No direct studies; marine benzofurans are under investigation for antioxidant and enzyme inhibitory effects relevant to diabetes.	Benzofuran derivatives can inhibit Î±-glucosidase and reduce oxidative stress in Î^2^-cells.
CMNPD26002	6-acetylbisdethiobis(methylthio)gliotoxin	Epipolythiodioxopiperazine	No direct anti-diabetic/GLP-1 studies	N/A
CMNPD5314	fumiquinazoline B	Quinazoline alkaloid	No direct studies for this compound; marine quinazoline alkaloids show anti-diabetic potential via enzyme inhibition and AMPK action.	Quinazoline alkaloids are highlighted for PTP1B inhibition and AMPK activation.
CMNPD22795	anthogorgiene I	Polycyclic aromatic (coral)	No direct anti-diabetic/GLP-1 evidence; marine polycyclic aromatics may have metabolic effects, but data are limited.	Structural novelty; potential for redox and enzyme modulation.
CMNPD27343	versiquinazoline B	Quinazoline alkaloid	No direct anti-diabetic/GLP-1 studies	N/A
CMNPD24593	speradine E	Alkaloid (marine fungus)	No direct studies	Alkaloids from marine origin reported with anti-diabetic activities

**Table 6:** ADMET profiling of the 10 hits from the CMNPD database.

ADMET Parameters	1	2	3	4	5	6	7	8	9	10	Control
**Absorption**											
Water solubility (log mol/L)	−3.506	−3.489	−2.791	−2.998	−3.011	−0.851	−3.745	−3.583	−3.32	−3.005	−2.96
Caco2 permeability (log Papp in 10^−6^ cm/s)	1.135	1.198	−0.546	0.151	0.994	1.23	−0.003	0.829	1.158	−0.305	0.986
Intestinal absorption (human) (% Absorbed)	97.322	96.618	3.62	48	95	100	54	96	100	48	63.055
P-glycoprotein substrate (Yes/No)	Yes	Yes	No	Yes	Yes	Yes	No	Yes	Yes	Yes	Yes
**Distribution**											
BBB permeability (log BB)	−0.043	0.228	−1.479	−1.378	−0.133	−0.398	−1.031	0.058	−0.485	−1.047	−1.635
CNS permeability (log PS)	−2.209	−2.002	−3.698	−2.508	−2.361	−3.778	−3.464	−1.894	−2.565	−2.895	−3.746
**Metabolism**											
CYP2D6 substrate (Yes/No)	No	No	Yes	Yes	No	No	No	No	No	Yes	No
CYP3A4 substrate (Yes/No)	Yes	Yes	No	No	No	Yes	No	Yes	Yes	No	No
CYP1A2 inhibitor (Yes/No)	Yes	Yes	No	Yes	Yes	No	No	Yes	Yes	No	No
CYP2C19 inhibitor (Yes/No)	No	No	No	No	Yes	Yes	No	No	No	No	No
CYP2C9 inhibitor (Yes/No)	No	No	No	Yes	No	Yes	No	No	No	No	No
CYP2D6 inhibitor (Yes/No)	No	Yes	No	No	Yes	No	No	No	No	No	No
CYP3A4 inhibitor (Yes/No)	No	No	No	No	Yes	Yes	No	No	Yes	No	No
**Excretion**											
Total Clearance (log mL/min/kg)	1.003	1.189	1.021	0.497	1.11	0.763	0.099	1.1	0.928	0.844	0.664
Renal OCT2 substrate (Yes/No)	No	No	No	No	Yes	No	No	Yes	No	No	No
**Toxicity**											
AMES toxicity (Yes/No)	Yes	Yes	No	No	No	No	No	Yes	No	No	No
Max. tolerated dose (human) (log mg/kg/day)	−0.349	−0.779	0.31	0.742	−0.154	0.528	0.595	0.122	−0.647	0.62	0.664
hERG I inhibitor (Yes/No)	No	No	No	No	No	No	No	No	No	No	No
Hepatotoxicity (Yes/No)	Yes	Yes	Yes	Yes	No	Yes	No	Yes	Yes	Yes	Yes

**Table 7:** Physicochemical properties, drug-likeness, and medicinal chemistry prediction of 10 hits from the CMNPD database.

Molecule Properties	1	2	3	4	5	6	7	8	9	10	Control
**Physicochemical properties**											
Molecular Weight	351.42	349.40	397.38	404.42	336.32	417.41	354.31	344.36	389.43	333.34	543.59
LogP	1.98	2.08	0.68	2.04	2.11	3.22	1.63	2.65	2.07	1.69	1.96
#Acceptors	3	3	7	6	5	7	7	3	4	6	8
#Donors	2	1	5	4	2	2	3	1	1	3	1
#Heavy atoms	26	26	29	30	25	31	26	26	29	24	40
#Arom. heavy atoms	13	13	9	20	18	22	12	19	20	10	21
Fraction Csp3	0.43	0.33	0.20	0.13	0.11	0.12	0.21	0.14	0.18	0.35	0.33
#Rotatable bonds	2	2	8	8	1	6	2	2	6	7	10
Molar refractivity	101.04	100.49	108.21	111.41	93.35	114.92	88.51	99.10	112.34	88.21	149.31
TPSA (Å^2^)	66.20	55.20	152.08	115.56	72.25	109.86	121.13	69.03	90.57	116.84	117.53
**Drug-likeness**											
Lipinski alert	yes	yes	no	yes	yes	yes	yes	yes	yes	Yes	yes
Ghose	yes	yes	no	yes	yes	yes	yes	yes	yes	Yes	no
Veber	yes	yes	no	yes	yes	yes	yes	yes	yes	Yes	Yes
Egan	yes	yes	no	yes	yes	yes	yes	yes	yes	Yes	Yes
Muegge	yes	yes	no	yes	yes	yes	yes	yes	yes	Yes	Yes
Bioavailability Score	0.55	0.55	0.55	0.55	0.55	0.55	0.56	0.55	0.55	0.56	0.55
**Medicinal chemistry**											
PAINS	0	0	0	0	1	0	1	0	0	0	0
Brenk	1	1	0	0	3	0	0	1	2	1	0
Synthetic accessibility	3.99	4.36	4.61	3.70	2.68	4.06	3.88	3.00	2.94	3.18	4.75

## Data Availability

All data generated or analyzed during this study are included in this published article.

## Supplementary Materials

The following supporting information can be downloaded at: https://www.mdpi.com/article/10.3390/ijms26136131/s1.
