# Supplementary material for: Structure-Based Discovery of Orthosteric Non-Peptide GLP-1R Agonists via Integrated Virtual Screening and Molecular Dynamics
Source: Int J Mol Sci. 2025 Jun 26;26(13):6131. doi: 10.3390/ijms26136131 (PMC12249819; doi:10.3390/ijms26136131)
Supplement: Supplementary file 1 [file ijms-26-06131-s001.zip › ijms-3689167-supplementary.pdf]

Molecular Dynamic Simulation Findings:

Molecular Dynamic (MD) simulation studies are critical towards the validation of the stability and strength of the ligand-protein binding interaction over a period of 100 nano seconds. Candidates for molecular dynamic simulation studies were shortlisted based on binding affinity and energy scores, as well as visual analysis of the binding interaction profiles. ligands with the highest complementary binding and energy scores, that were shown to bind with key residues in the GLP-1 binding pocket were selected. Figure S1 highlights representative ligands from the marine database which exhibited a robust and stable binding pattern within the binding pocket of the GLP-1 receptor. RMSD plot analysis was used to measure the average displacement of atoms for a particular frame with respect to a reference frame of the ligand bound within the protein pocket. MD calculation analysis revealed a stable binding profile, as indicated by an RMSD fluctuation range within 2-6 Å of the protein backbone and the ligand throughout the simulation period. Figure S3 shows representative RMSD plots for two ligands: Hit **11**: CMNPD27661 and Hit**13**: CMNPD24592 , from the comprehensive marine natural products database. Figures S1 and S2 illustrate and describe the detailed binding interaction analysis for Hit **11**: CMNPD27661 and Hit**13**: CMNPD24592 Hitrespectively.

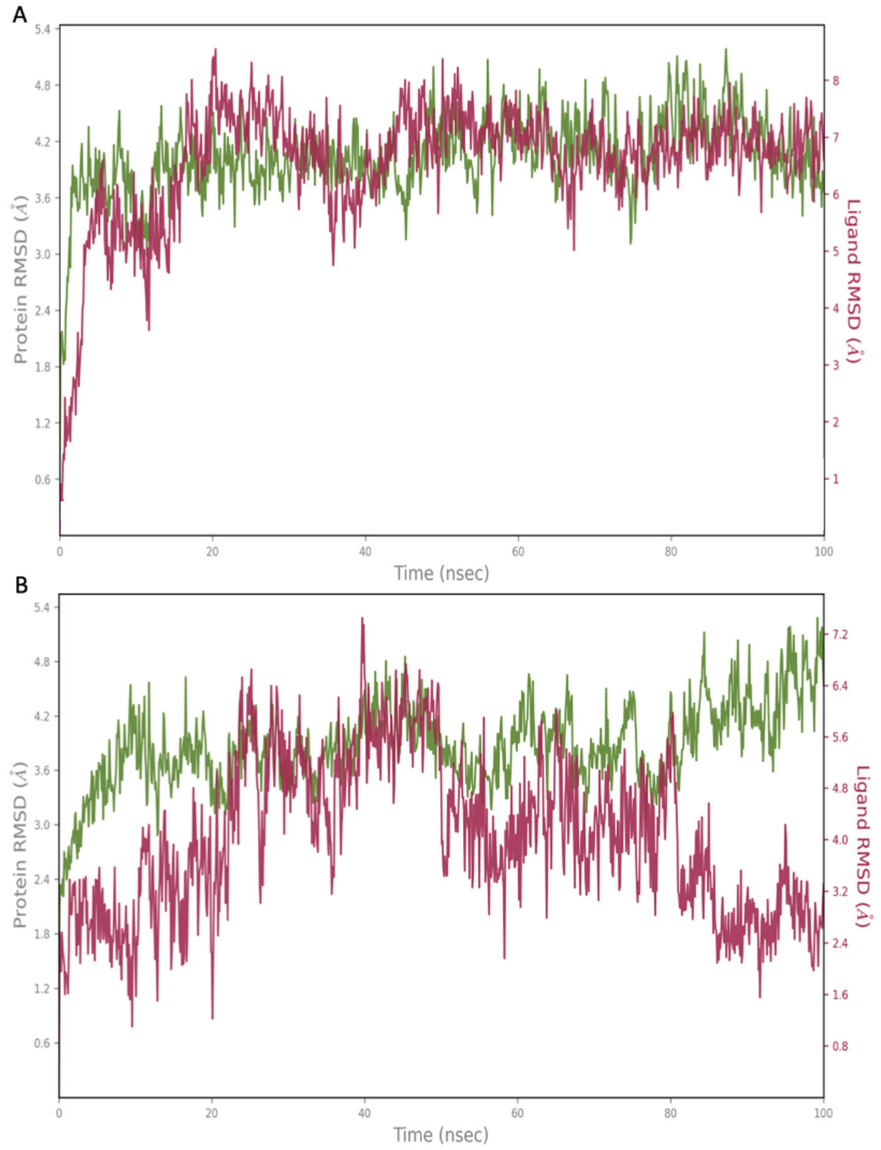

Figure S1: Root mean square deviation (RMSD) graphs for the top hit compounds: ([illegible]) Hit**13**: CMNPD24592, (B) Hit **11**: CMNPD27661. The green graph shows the fluctuations in the protein backbone from the initial reference point, while the red shows the ligand fluctuations. The RMSD profile of the ligand is with respect to its initial fit to the protein binding pocket indicates that all ligands, fluctuation range is within a 2–6 Å range for the majority if the simulation period.

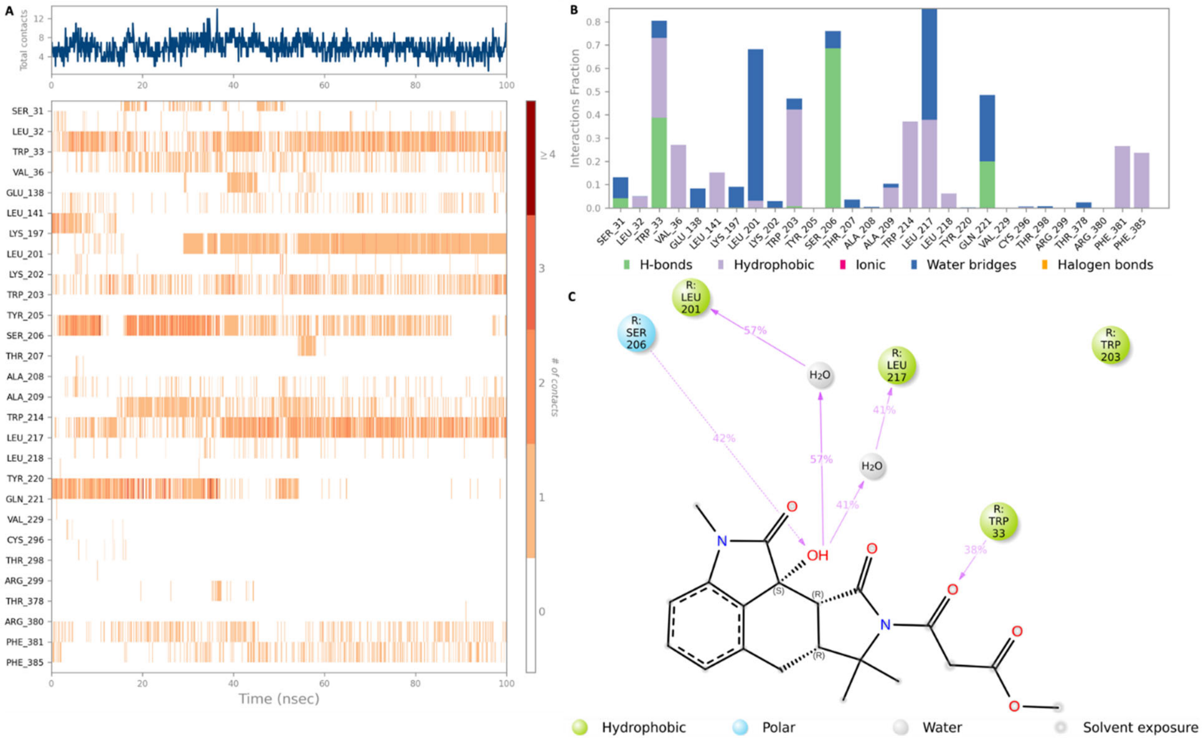

Figure S2: Interaction diagram of compound **13** within the GLP-1 protein binding pocket. (□) Interaction of Hit **13** CMNPD24592 with residues in each trajectory frame. The depth of colour reflects the strength of the interaction and contacts with the residues; (B) the protein–ligand contacts showing the bonding interaction fractions and the nature of the interactions; (C) graphical 2D illustration of compound **13** interacting with the protein residues during MD simulation. Interactions shown occurred over 30% of the simulation time.

Figure S2 shows the binding profile of Hit **13** CMNPD24592 with residues of the GLP-1 receptor binding pocket. Key binding residues TRP-203 and PHE-381 form hydrophobic interactions with the ligand. Yet the strongest interactions are observed between residues SER-206, LEU-201 and LEU-217 in the receptor binding pocket with hydroxyl group of the hit compound. □ strong hydrogen bonding interaction is observed at SER 206 for 42% of the simulation period. Whereas a combination of water bridge and hydrophobic interactions are observed at LEU-201 and LEU-217 for 57% and 41% of the molecular dynamic simulation period. Significant hydrogen bonds were also observed with other binding residues, namely TRP-33 and GLN-221.

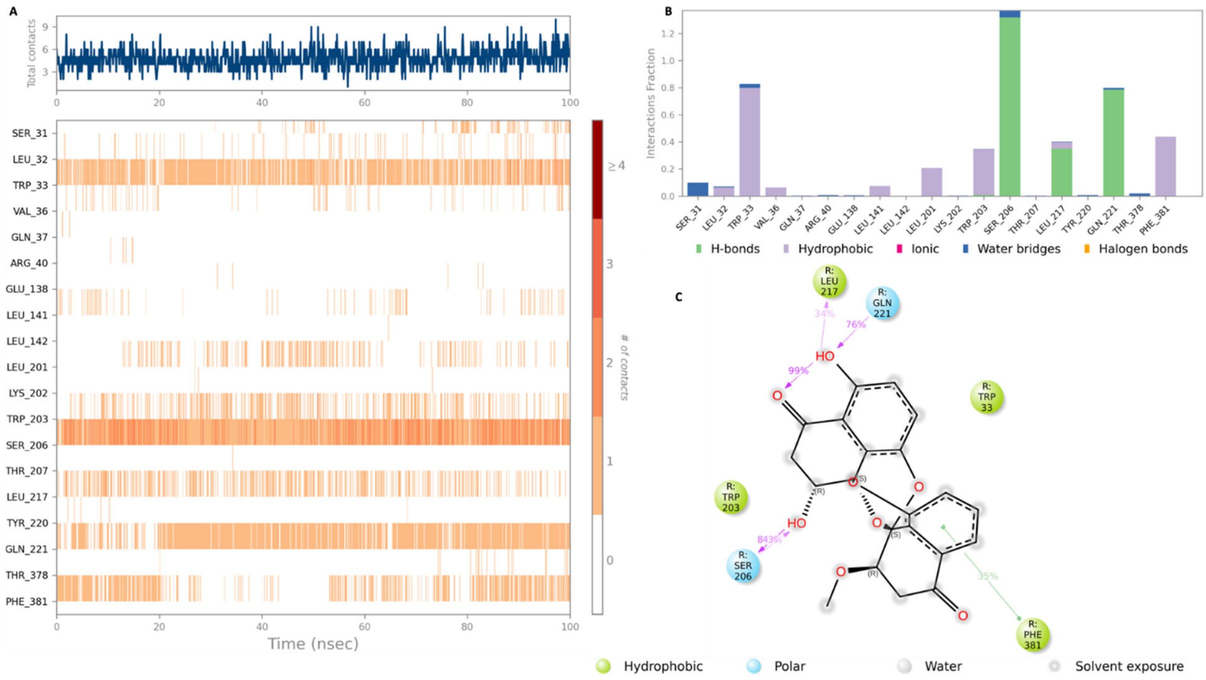

Figure S3: Interaction diagram of compound **11** within the GLP-1 protein binding pocket. ([illegible]) Interaction of Hit **11** CMNPD27661 with residues in each trajectory frame. The depth of colour reflects the strength of the interaction and contacts with the residues; (B) the protein–ligand contacts showing the bonding interaction fractions and the nature of the interactions; (C) graphical 2D illustration of compound **11** interacting with the protein residues during MD simulation. Interactions shown occurred over 30% of the simulation time.

The MD simulation results for Hit **11** CMNPD27661 in Figure S3 shows moderate hydrophobic interactions with the key binding residues TRP 203 and PHE-381 in the GLP-1 binding pocket. The interaction with PHE-381 is observed for 35% of the simulation period. Of significance, a strong hydrogen bond interaction is observed between SER 206 and the hit ligand for 84% of the simulation period. [illegible]nother hydrogen bond interaction is observed at GLN 221 and LEU 217 for 76% and 34% of the MD simulation period. Once more, for this marine-derived compound, a key interaction is observed at TRP 33 in the form of a hydrophobic interaction. This interaction remains stable throughout the majority of the simulation period, based on the number of contacts illustrated in Figure S3 ([illegible]).
